# Epidemiology, clinical management, and outcomes in patients with eosinophilic granulomatosis with polyangiitis in England: A retrospective observational cohort study

**DOI:** 10.1016/j.jacig.2026.100740

**Published:** 2026-06-01

**Authors:** Salman Siddiqui, Bo Ding, Paul Dolin, Chris Edmonds, Priya Jain, Jennifer Rowell, Lotte Westerink, Alessandra Lacetera, Pablo Suárez-Sánchez, Cono Ariti, Bélène Podmore, Alvaro Kitchin Velarde, Stephanie Y. Chen

**Affiliations:** a National Heart and Lung Institute, National Institute for Health and Care Research Imperial Biomedical Research Centre, Imperial College London; b BioPharmaceuticals Medical, AstraZeneca, Gothenburg, Mölndal, Sweden; c BioPharmaceuticals Medical, AstraZeneca, Cambridge, United Kingdom; d Market Access and Pricing, AstraZeneca, Gaithersburg, Md; e Health Economics and Payer Evidence, AstraZeneca, Cambridge, United Kingdom; f OXON Epidemiology, Madrid, Spain; g BioPharmaceuticals Medical, AstraZeneca, Gaithersburg, Md

**Keywords:** Eosinophilic granulomatosis with polyangiitis, EGPA, epidemiology, diagnosis, mortality, survival, treatment, remission, relapse

## Abstract

**Background:**

Data on the clinical burden of eosinophilic granulomatosis with polyangiitis (EGPA) are limited.

**Objective:**

We sought to evaluate the epidemiology and clinical burden of EGPA in England using real-world evidence.

**Methods:**

Patients diagnosed with EGPA between January 1, 2006, and February 28, 2019, who had ≥1 year of data before diagnosis (index date) were identified using the Clinical Practice Research Datalink Aurum database. Epidemiology, diagnosis, mortality, treatment, and clinical outcomes were assessed.

**Results:**

The incident and prevalent EGPA cohorts comprised 486 and 729 patients, respectively. The overall incidence and prevalence of EGPA were 3.04 (95% CI: 2.77-3.32) cases per million person-years and 2.7 (95% CI: 2.5-2.9) cases per 100,000 persons, respectively. Overall, 76.3% and 26.1% of patients had a Five Factor Score of 0 on the 1996 and 2009 versions. In the incident cohort (mean age 57.9 ± 15.2 years), most patients (97.1%) had ≥1 comorbidity; 79.8% had asthma coded. The median time from first major manifestation to EGPA diagnosis was 44.0 (Q1-Q3: 20.0-56.0) months. The death rate was 37.1 per 1000 person-years (95% CI: 30.1-45.2); the standardized mortality ratio for all-cause deaths was 2.3 (95% CI: 1.9-2.8). The 5-year survival rate was 82.3% (95% CI: 78.1%-85.7%). Most patients (86.2%) received oral glucocorticoids, of whom 27.0% successfully tapered. Six months post index date, 26.1% of patients had a new EGPA manifestation.

**Conclusion:**

This study emphasizes the substantial clinical burden and reliance on glucocorticoids in EGPA, highlighting the need for improved diagnosis of this disorder.

Eosinophilic granulomatosis with polyangiitis (EGPA) is a rare chronic immune disorder characterized by small-to-medium–size vessel necrotizing vasculitis, eosinophilic granulomatous inflammation, and adult-onset asthma.^1^^,^^2^ EGPA has an estimated global incidence and prevalence of 0.5 to 4 cases per million person-years (PY) and 14-59 cases per million people, respectively, depending on geography and diagnostic criteria.^3^, ^4^, ^5^, ^6^, ^7^, ^8^, ^9^, ^10^ A retrospective longitudinal study in patients in the United Kingdom with EGPA reported a prevalence of 22.7 to 45.6 cases per million people (2005-2019), and an incidence of 2.3 to 4.0 per million PY (2006-2019).^5^ A meta-analysis of real-world observational studies (January 1, 2019–June 6, 2023) has estimated that EGPA has a mortality rate of 10.63 (95% CI: 7.20-15.71) per 1000 PY.^9^

The disease's clinical burden is substantial,^4^^,^^5^^,^^8^ with multisystemic involvement,^8^^,^^11^ including the cardiovascular,^12^ gastrointestinal,^13^ nervous,^14^ and renal^15^ systems. EGPA has a relapsing and remitting disease course, with many patients experiencing relapse even after achieving remission.^1^, ^2^, ^3^ A relapse frequency of 25% to 49% is reported in the literature,^16^, ^17^, ^18^ and the principal aim of treatment is to reduce the risk of relapse and induce long-term remission. Treatment primarily involves long-term oral glucocorticoids (OGCs), often combined with immunosuppressants and/or biologics where approved.^19^, ^20^, ^21^, ^22^, ^23^ Due to the risks of prolonged OGC use, tapering is recommended,^19^, ^20^, ^21^^,^^24^ although relapses often occur during this process.^16^^,^^25^^,^^26^

Real-world data on EGPA epidemiology and clinical burden are limited, underscoring the need for further studies. A retrospective study using the Clinical Practice Research Datalink (CPRD) Aurum database found stable EGPA incidence in England from 2005 to 2019, increasing prevalence, and consistently high health care resource and OGC use.^5^ Here, we expanded on these findings by including the patient journey, mortality and survival by Five Factor Score (FFS), manifestations by Birmingham Vasculitis Activity Score (BVAS) items, and transitions between disease states (remission, stable disease, and relapse).

## Methods

### Study design

This was a retrospective observational cohort analysis that used data from the CPRD Aurum primary care database, which is linked to the Hospital Episodes Statistics inpatient, outpatient, and accident and emergency records and the Office for National Statistics mortality data from England (Independent Scientific Advisory Committee approved study protocol number: 22_001934). The patient inclusion period was from January 1, 2006, to February 28, 2019, with follow-up until February 28, 2020 (see Fig E1 in this article’s Online Repository at www.jaci-global.org). A look-back period of up to 5 years prior to the date of the patient's first EGPA diagnosis (referred to throughout as the “index date” [ID]) was established, with ≥1 year prior to 2006 or ≥1 year prior to ID to differentiate incident and prevalent cases.

The study included an incident cohort with a first ID of EGPA based on Systematized Nomenclature of Medicine Clinical Terms (SNOMED CT) 82275008 or International Classification of Diseases, Tenth Revision, Clinical Modification [ICD-10-CM] M30.1 clinical codes recorded from January 1, 2006, to February 28, 2019 (see Table E1 in this article’s Online Repository at www.jaci-global.org). Patients were included if they had ≥1 year of medical records before and including ID, contributed ≥1 day in the CPRD database after ID, and did not have any diagnosis of the following diseases: Wegener's granulomatosis or granulomatosis with polyangiitis, microscopic polyangiitis, polyarteritis nodosa, Takayasu arteritis, or giant cell arteritis.

This study forms part of the CONSTELLATION real-world evidence program focused on rare eosinophil-associated disease.

### Outcomes

#### Baseline demographics and clinical characteristics

Prognosis was evaluated using the FFS 1996 and 2009 versions (see the Methods in this article’s Online Repository at www.jaci-global.org for the parameters assessed), using the relevant SNOMED CT and ICD-10 codes (see Table E2 in this article’s Online Repository at www.jaci-global.org) up to 5 years pre-ID (except age >65 years). FFS scores were 0 if none of the codes for the clinical factors were present, 1 if codes for 1 factor were present, and 2 if codes for ≥2 of the factors were present, with lower scores associated with better prognosis.

History of comorbidities was assessed at ID using code lists containing the relevant SNOMED + ICD-10 codes (see Table E3 in this article’s Online Repository at www.jaci-global.org). Once identified, these comorbidities were considered as being present thereafter.

#### Incidence and prevalence

The EGPA annual incidence and the prevalence observed from 2006 to 2019 were assessed overall and by year, age, and sex (see the Methods in this article’s Online Repository for further details and calculations).

#### Patient journey

The number of visits to specialists and medical specialties were assessed, and the specialists with the highest number of visits for each patient at 12 months pre- and post-ID were identified.

#### Mortality and survival

The number of deaths and overall survival during follow-up were assessed from 2006 to 2020. These were assessed overall, over time, and by FSS (1996 and 2009 versions), age, and sex. The mortality rate and standardized mortality ratio (SMR) were calculated. Further details, including SMR calculations, are reported in the Methods in this article’s Online Repository.

#### Treatment patterns

Medication use was derived from primary care records only, using CPRD Aurum ProdCodes mapped to the Dictionary of Medicines and Devices codes. A prescription of EGPA medication was defined using relevant product codes. Treatment discontinuation was defined as a gap of 60 days without a repeat prescription for the drug of interest. Daily dose was calculated as the product of quantity and substance strength, divided by the prescription estimated duration. Medication exposure episode was defined as the period from the first prescription date or the first prescription following a gap of ≥60 days until discontinuation.

Prescribing rates (post-ID), duration of therapy, medication regimens, OGC and immunosuppressant dosage (pre- and post-ID), and OGC tapering (post-ID) were assessed (see the Methods in this article’s Online Repository). Medication regimens (defined as any medication prescribed for the treatment of EGPA either in monotherapy or in combination) were assessed 1 year pre-ID, over the entire post-ID period, and within specified study periods.

OGC tapering refers to the gradual reduction or discontinuation of the OGC dose over time. The criteria for qualifying for tapering are detailed in the Methods in this article’s Online Repository.

#### Clinical outcomes

EGPA manifestations were assessed based on items reported in the database equivalent to those included in the BVAS version 3.^27^ Patients were considered to have an EGPA manifestation (major or nonmajor) if they had a relevant SNOMED CT or ICD-10 code recorded for any of the conditions listed in Table E4 in this article’s Online Repository at www.jaci-global.org, which were considered related to EGPA. New EGPA manifestations (first-ever occurrence with no previous EGPA event in the available data history up to 5 years pre-ID) were assessed in the 6 months pre-ID and during follow-up.

Asthma exacerbations were measured in the 6 months before ID and during follow-up. Patients were considered to have new-onset asthma if the first diagnosis of asthma occurred post-ID. Organ damage was assessed at ID and 6-month intervals post-ID, using the diagnostic codes associated with the items of the Vasculitis Damage Index^28^ (see Table E5 in this article’s Online Repository at www.jaci-global.org), regardless of the cause.

EGPA disease status was assessed at ID and during follow-up, every 6 months for 2 years with a 6-month look-back period at each time point, and then annually for years 3-5 with a 12-month look-back period at each time point. At each assessment, patients were categorized into 1 of 3 mutually exclusive categories: remission, relapse, or stable disease. Remission was defined as no new EGPA manifestation (Table E4), no OGC use or OGC of ≤4 mg/day, no initiation of immunosuppressant or cyclophosphamide, and no EGPA-related hospitalization. Relapse was defined as a new EGPA manifestation or EGPA-related hospitalization, and any systemic corticosteroid initiation, reinitiation of >4 mg/day or increase of >4 mg/day or initiation of immunosuppressant or cyclophosphamide. Types of relapses included asthma, nasal polyposis, vasculitic, and EGPA hospitalization (see the Methods in this article’s Online Repository). Stable disease was defined as not in remission and not in relapse (eg, all OGC doses >4 mg/day or only last dose of the time period <4 mg/day).

The statistical analyses are described in the Methods in this article’s Online Repository.

## Results

### Baseline demographics and clinical characteristics

A total of 2349 patients with EGPA were identified in the database, of which 729 patients were included in the prevalence estimation, and 486 patients in the incident EGPA cohort (see Fig E2 in this article’s Online Repository at www.jaci-global.org). The mean age at ID was 57.9 ± 15.2 years, with 71.8% of patients >50 years old and only 1% <18 years; 50.2% of patients were female, 88.3% were White, and 25.7% had an Index of Multiple Deprivation quintile of 1 (least deprived). The mean follow-up was 5.4 ± 3.7 years (see Table E6 in this article’s Online Repository at www.jaci-global.org).

#### FFS

Using the 1996 version, the majority of patients (76.3%) had a score of 0, 20.4% had a score of 1, and 3.3% had a score of ≥2. Using the 2009 version, 26.1% of patients scored 0, while the majority scored either 1 (44.7%) or ≥2 (29.2%).

#### Comorbidities

**Fig 1:**
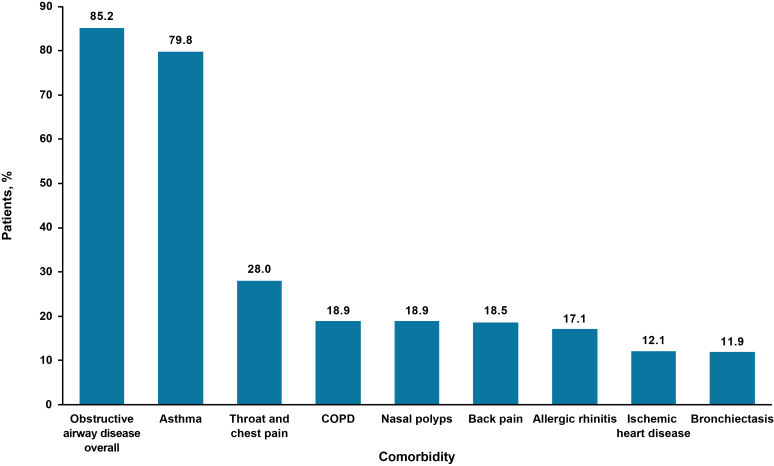
Comorbidities at ID in the EGPA incident cohort. Comorbidities with incidence >10% have been included. *COPD*, Chronic obstructive pulmonary disease.

Among the EGPA cohort, 97.1% of patients had ≥1 comorbidity; obstructive airway disease affected 85.2% of patients, with asthma of all severity being the most commonly coded (79.8%), followed by chronic obstructive pulmonary disease (18.9%) and bronchiectasis (11.9%) (Fig 1).

### Incidence and prevalence from 2006 to 2019

**Fig 2:**
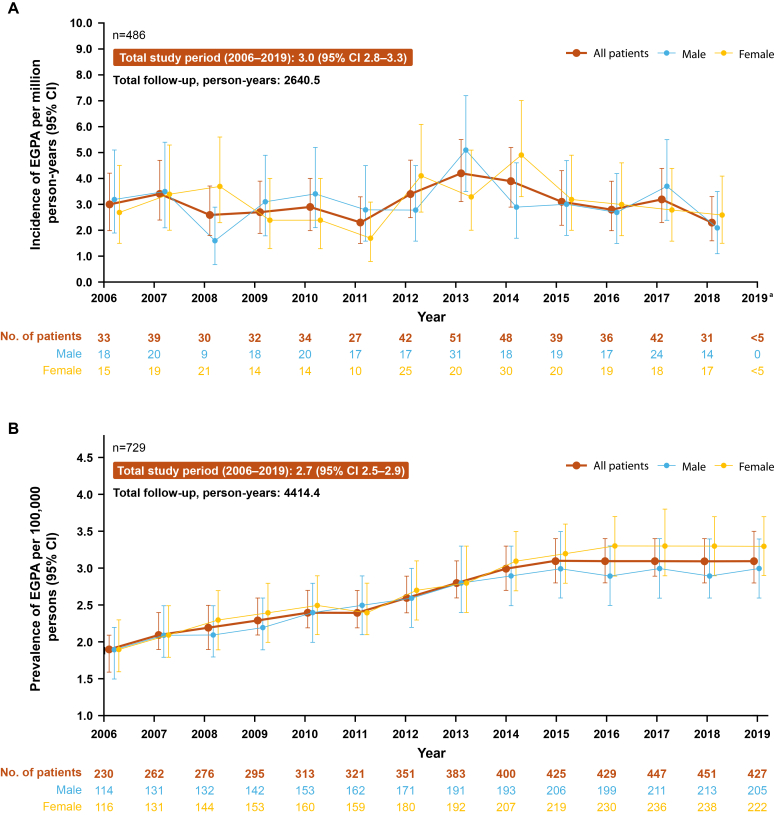
Incidence and prevalence of EGPA during the study period. **(A)** Incidence and **(B)** prevalence of EGPA during the study period (2006-2019). n = prevalent cases. ^a^The follow-up ended on February 28, 2020, but the recruitment ended on February 28, 2019. The prevalence estimates were calculated without taking into consideration the 12-month look-back period before and inclusive of the ID.

The overall incidence of EGPA diagnosis was 3.04 (95% CI: 2.77-3.32) cases per million PY. The incidence did not vary significantly across the study period, from 3.0 (95% CI: 2.0-4.2) in 2006 to 2.3 (95% CI: 1.6-3.3) in 2018; range 2.25 (95% CI: 1.48-3.28) to 4.19 (95% CI: 3.12–5.51) per million PY. There were no significant differences by sex (Fig 2, *A*).

The overall prevalence of EGPA was 2.7 (95% CI: 2.5-2.9) cases per 100,000 persons. The prevalence steadily increased from 1.9 (95% CI: 1.6-2.1) in 2006 to 3.1 (95% CI: 2.8-3.4) in 2015, remaining at 3.1 (95% CI: 2.8-3.5) through to 2019. There were no significant differences by sex (Fig 2, *B*).

### Patient journey

**Fig 3:**
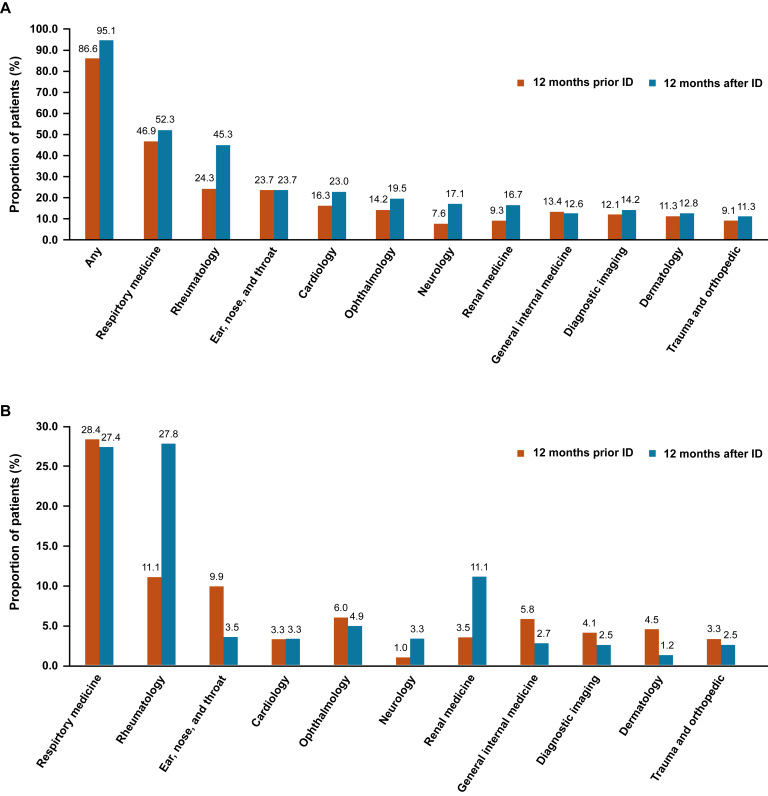
Proportion of patients visiting specialists. Proportion (%) of patients **(A)** with any visit to a specialist by type and **(B)** visiting the most frequently consulted specialist per patient.

Based on the 85.6% of patients with a major EGPA symptom in the 5 years pre-ID, the median time from first major manifestation to EGPA diagnosis, including asthma diagnosis or nasal polyposis, was 44.0 (Q1-Q3: 20.0-56.0) months. In the 12 months pre-ID, 86.6% of patients had ≥1 visit to a specialist, most commonly to respiratory medicine (46.9%), rheumatology (24.3%), and ear, nose, and throat (ENT) services (23.7%). In the 12 months post-ID, the proportion of patients with ≥1 specialist visit increased to 95.1% (Fig 3, *A*). When considering the specialists with the highest number of visits for each patient, respiratory specialists were the most frequently consulted physicians for 28.4% of patients in the 12 months pre-ID, followed by rheumatologists (11.1%) and ENT specialists (9.9%) (Fig 3, *B*). In the 12 months post-ID, 27.8% of patients more frequently consulted rheumatologists, followed by respiratory specialists (27.4%) and ENT Service (3.5%) (Fig 3, *B*).

### Mortality and survival

**Fig 4:**
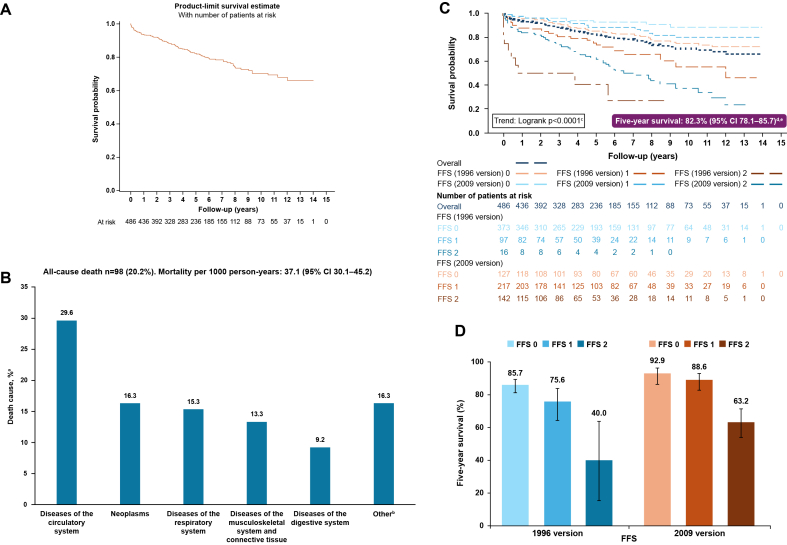
Survival probabilities and rates and causes of death. **(A)** Survival probability; **(B)** primary cause of death; **(C)** survival probability by FFS; and **(D)** 5-year survival rates by FFS. ^a^The percentages use all-cause death (n = 98) as the denominator. ^b^Other category includes unknown, external causes of morbidity and mortality; III. Diseases of the blood and blood-forming organs and certain disorders involving the immune mechanism; XIV. Diseases of the genitourinary system; IV. Endocrine, nutritional and metabolic diseases; I. Certain infectious and parasitic diseases, XII. Diseases of the skin and subcutaneous tissue, and V. Mental and behavioral disorders. ^c^FFS 1996 and 2009 versions. ^d^By index year: 2006 to 2010: 78.7% (95% CI: 71.4%-84.4%); 2011 to 2015: 85.7% (95% CI: 79.8%-90.0%); 2016 to 2019: not available. ^e^Five-year survival was greater when using criteria for the FFS 2009 version than the FFS 1996 version, and for patients with index year between 2011 and 2015 than between 2006 and 2010.

A total of 98 deaths (20.2%) were observed in the incident EGPA cohort (median follow-up of 4.8 years), corresponding to a death rate of 37.1 per 1000 PY (95% CI: 30.1-45.2). The SMR for all-cause deaths was 2.3 (95% CI: 1.9-2.8). At 5 years, the survival rate was 82.3% (95% CI: 78.1-85.7) (Fig 4, *A*).

The most common causes of death included EGPA (11.2%), chronic ischemic heart disease (6.1%), lung malignancies (6.1%), and acute myocardial infarction (5.1%). Stratification by ICD-10 chapters showed that the leading cause of death was circulatory/cardiovascular diseases (29.6%), followed by neoplasms (16.3%), respiratory diseases (15.3%), and musculoskeletal/connective tissue diseases (13.3%) (Fig 4, *B*). When stratified by index year, the highest proportion of deaths (52.0%) occurred in the 2006-2010 cohort, with a death rate of 39.0 per 1000 PY (see Table E7 in this article’s Online Repository at www.jaci-global.org).

Patients with an FFS 1996 score of 0 had the lowest mortality rate (28.3 per 1000 PY), whereas those with FFS 1996 scores of 1 and 2 or more experienced markedly higher rates (61.2 and 239.0 per 1000 PY, respectively). Similar results were obtained when using the FFS 2009 (Table E7). Survival decreased with increasing FFS score and was greater when assessed using the 2009 version compared to the 1996 version (Fig 4, *C* and *D*). Stratification by health state at ID revealed that patients in remission had a lower SMR of 1.50 (95% CI: 0.82-2.52) compared with those not in remission (SMR: 2.55; 95% CI: 2.04-3.16) (see Table E8 in this article’s Online Repository at www.jaci-global.org).

### Treatment patterns

Across the study period, OGCs were the most frequently prescribed medications in primary care, with 86.2% of patients receiving OGCs (prednisolone), followed by immunosuppressants (53.7%). Azathioprine (32.5%) was the most prescribed immunosuppressant (Table I). Among patients prescribed OGCs post-ID, the overall median daily dose was 14.3 mg/day (minimum [min], maximum [max]: 3.0, 50.9), with a median cumulative dose for all follow-up (median follow-up 4.8 years) of 399.4 mg (min, max: 5.02, 2690.9) (Table II).

**Table I:** EGPA-related drug prescribing rates in primary care in the incident EGPA cohort (n = 486) at any time after ID

	Patients with a prescription, n (%)	Prescription rate per PY (95% CI)
Immunosuppressants	261 (53.7)	7.3 (7.2-7.4)
Azathioprine	158 (32.5)	6.5 (6.4-6.7)
Cyclophosphamide	13 (2.7)	0.5 (0.4-0.7)
Mycophenolate mofetil	72 (14.8)	4.7 (4.5-4.9)
Methotrexate	85 (17.5)	5.7 (5.5-5.9)
OGCs	419 (86.2)	8.2 (8.1-8.3)
Prednisone	<5	—
Prednisolone	419 (86.2)	8.2 (8.1-8.3)
Methylprednisolone	0 (0.0)	—
IV/IM-corticosteroid	15 (3.1)	0.3 (0.2-0.4)
Prophylactic	0 (0.0)	—
Nonprophylactic	15 (3.1)	0.3 (0.2-0.4)

*IM*, intramuscular; *IV*, intravenous.

**Table II:** Estimation of OGC dose∗ used in primary care in the incident EGPA cohort (n = 486)

	Total post-ID	6 months prior ID	0–6 months post-ID
Daily dose (mg/day)			
Mean ± SD	15.8 ± 8.8	16.3 ± 12.6	21.8 ± 12.8
Median (min, max)	14.3 (3.0, 50.9)	12.4 (0.7, 75.0)	19.6 (2.3, 70.3)
Daily dose (mg/day) categories			
≤4.0	2 (0.5)	18 (6.0)	8 (2.1)
4.1-10.0	109 (26.0)	108 (36.0)	63 (16.7)
10.1-20.0	220 (52.5)	93 (31.0)	126 (33.4)
20.1-40.0	77 (18.4)	67 (22.3)	151 (40.1)
>40.0	11 (2.6)	14 (4.7)	29 (7.7)
Maximum daily dose (mg/day)			
n	419	300	377
Mean ± SD	54.2 ± 31.0	24.7 ± 21.3	35.3 ± 24.2
Median (min, max)	49.4 (5.0, 168.0)	17.9 (0.7, 100.0)	30.0 (2.5, 120.0)
Maximum daily dose (mg/day) categories			
≤4.0	0 (0.0)	14 (4.7)	5 (1.3)
4.1-10.0	18 (4.3)	84 (28.0)	41 (10.9)
10.1-20.0	36 (8.6)	71 (23.7)	91 (24.1)
20.1-40.0	117 (27.9)	82 (27.3)	124 (32.9)
>40.0	248 (59.2)	49 (16.3)	116 (30.8)
Cumulative dose (mg)			
n	419	300	377
Mean ± SD	569.2 ± 542.3	59.5 ± 74.7	101.7 ± 110.3
Median (min, max)	399.4 (5.0, 2690.9)	38.3 (0.7, 537.5)	73.4 (2.5, 839.5)

Data are n (%) unless stated otherwise.

∗ Prednisolone equivalent doses (1.25 for oral methylprednisolone, 1.53 for methylprednisolone injected, and 1 for prednisone).

The overall median duration of treatment episodes for OGCs was 2.0 (Q1-Q3: 1.0-4.6) months, with an average of 8.1 episodes per patient and an average annual number of episodes by patient of 1.4 (see Table E9 in this article’s Online Repository at www.jaci-global.org). For immunosuppressants, 54.3% of patients initiated ≥1 treatment episode of immunosuppressants in the overall pre- and post-ID. The median duration of these episodes was 1.5 (Q1-Q3: 1.0-2.8) months (Table E9).

Across the total post-ID period, prednisolone alone was the most frequently used regimen, documented in 37.4% of patients. Regimens that included immunosuppressants and prednisolone were also prominent (see Table E10 in this article’s Online Repository at www.jaci-global.org). During the total post-ID period, the median daily dose was 100.0 mg/day (min, max: 25.0, 316.7) for azathioprine, and 100.0 mg/day (min, max: 0.0, 186.2) for cyclophosphamide. Mycophenolate mofetil was administered at a median dose of 1,908.5 mg/day (min, max: 0.2, 125,000.1), and methotrexate at 2.9 mg/day (min, max: 1.2, 10,367.8).

Overall, 27.0% of patients successfully tapered their steroids, while 29.4% experienced partial tapering. Only 1 patient (0.4%) did not attempt tapering, and 43.3% were categorized as not tapered or having unsuccessful tapering outcomes.

### Clinical outcomes

#### EGPA manifestations

In the first 6 months post-ID, 127 patients (26.1%) experienced a new EGPA manifestation (Table III), with major EGPA manifestations observed in 9.7%. The most common of these were mononeuritis multiplex (2.7%), congestive heart failure (1.6%), and respiratory failure (1.4%). Overall, 20.8% of patients experienced nonmajor EGPA manifestations 6 months post-ID, with the most common being hypertension (2.7%), stage II-III chronic kidney disease (2.5%), and nasal polyposis (1.9%).

**Table III:** New EGPA manifestations at ID and during follow-up in the incident EGPA cohort

BVAS items	Time since ID (mo)		
	0-6	7-12	13-18
Patients at start of period	486	451	436
≥1 new manifestation	127 (26.1)	82 (18.2)	56 (12.8)
Major EGPA manifestations	47 (9.7)	22 (4.9)	10 (2.3)
Hemoptysis or alveolar hemorrhage	<5	<5	0 (0.0)
Respiratory failure	7 (1.4)	<5	0 (0.0)
Ischemic cardiac pain	<5	<5	<5
Cardiomyopathy	6 (1.2)	<5	<5
Congestive heart failure	8 (1.6)	<5	5 (1.1)
Hematuria	<5	<5	<5
Stage IV-V chronic kidney, end stage renal disease or renal dialysis	6 (1.2)	<5	0 (0.0)
Mononeuritis multiplex (without radiculopathy)	13 (2.7)	<5	<5
Nonmajor EGPA events	101 (20.8)	63 (14.0)	50 (11.5)
General	13 (2.7)	8 (1.8)	6 (1.4)
Myalgia	<5	<5	0 (0.0)
Arthralgia	7 (1.4)	5 (1.1)	6 (1.4)
Fever	<5	<5	0 (0.0)
Skin	5 (1.0)	<5	<5
Purpura	<5	0 (0.0)	<5
Urticaria	<5	<5	0 (0.0)
Ocular	7 (1.4)	6 (1.3)	5 (1.1)
Conjunctivitis, blepharitis or keratitis	<5	<5	<5
ENT	20 (4.1)	10 (2.2)	10 (2.3)
Nasal polyposis	9 (1.9)	<5	<5
Acute or chronic sinusitis	7 (1.4)	7 (1.6)	7 (1.6)
Chest	18 (3.7)	16 (3.5)	<5
Asthma	6 (1.2)	<5	<5
Wheeze	<5	5 (1.1)	0 (0.0)
Lung nodules or cavities	<5	<5	<5
Pleural effusion, pleurisy or pleuritis	5 (1.0)	<5	0 (0.0)
Endobronchial involvement	5 (1.0)	<5	0 (0.0)
Cardiovascular	<5	<5	<5
Valvular heart disease	<5	<5	<5
Renal	28 (5.8)	17 (3.8)	14 (3.2)
Hypertension	13 (2.7)	10 (2.2)	6 (1.4)
Proteinuria	6 (1.2)	<5	<5
Stage II-III chronic kidney disease or unspecified	12 (2.5)	<5	<5
Nervous system	16 (3.3)	8 (1.8)	8 (1.8)
Headache	7 (1.4)	6 (1.3)	6 (1.4)
Sensory peripheral neuropathy	5 (1.0)	<5	<5

Values are n or n (%).

#### Asthma exacerbations

In the 6 months pre-ID, 13.0% of patients experienced asthma exacerbations, with 8.4% having 1 and 4.5% having >1 exacerbation. Following ID, the proportion of patients with exacerbations declined to 6.2% in the 6 months post-ID and remained relatively stable thereafter.

#### Organ damage

In the first 6 months post-ID, 14 patients (2.9%) experienced persistent damage in ≥1 organ, increasing to 81 (28.6%) in the 49- to 60-month period post-ID. At 49 to 60 months post-ID, the organ damage predominantly affected the ocular (6.7% with cataracts), ENT (6.4%), cardiovascular (5.7%), pulmonary (4.6%), and musculoskeletal (4.6%) systems (see Table E11 in this article’s Online Repository at www.jaci-global.org).

#### Health state

**Fig 5:**
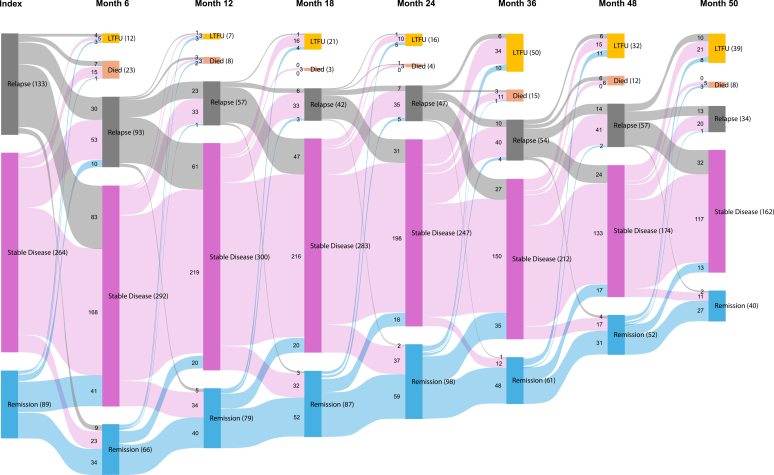
EGPA-related health state transitions at ID and follow-up. Data are reported in number of patients. *LTFU*, Lost to follow up.

At ID, 89 patients (18.3%) were in remission, 264 (54.3%) had stable disease, and 133 (27.4%) had relapsed. The most common type of relapse at any point during follow-up was vasculitic relapse (78.6%-89.5%), followed by EGPA hospitalization (30.1%-35.3%). Over time, stable disease remained the most common state, while fewer patients achieved or maintained remission (Fig 5). Excluding patients who died or were lost to follow-up, among patients in remission at ID (n = 85), 34 (40.0%) maintained remission at 6 months. Among those not in remission at ID (n = 366), 32 (8.7%) achieved remission by 6 months.

## Discussion

This is the first population-level comprehensive assessment of patients with EGPA characterizing mortality and survival by FFS, disease manifestations based on BVAS items,^27^ treatment utilization, and transitions between disease states. Furthermore, it includes one of the largest EGPA cohorts reported.^5^^,^^8^^,^^17^^,^^29^^,^^30^

Most patients (79.8%) had comorbid asthma of any severity, consistent with EGPA’s known profile, where most patients, although not all, have asthma. In contrast, the frequencies of chronic obstructive pulmonary disease and bronchiectasis were higher than expected.^1^^,^^2^^,^^31^^,^^32^ The estimated incidence and prevalence reported in this study align with previous data on the rarity of EGPA.^1^^,^^2^ Incidence rates in our study (range 2.3-4.2 cases per million PY) were relatively stable over time and similar to those reported over the same time period (2006-2019) by Hwee et al^5^ in another analysis based on CPRD Aurum data (range 2.3-4.0 cases per million PY). This suggests diagnostic practices and disease awareness may have remained consistent, although subtle changes in health care access or coding practices cannot be excluded. Two systematic literature reviews reported an EGPA incidence of 2.2 cases per million PY in the United Kingdom^9^ and 1.1 cases per million PY in Europe.^3^ We observed an increase in EGPA prevalence from 2006 until 2015 (from 19 to 31 cases per million people), after which prevalences stabilized. In contrast, Hwee et al^5^ reported a steady increase through 2019 (from 22.7 to 45.6 cases per million people from 2005 to 2019). The reasons for this discrepancy are unclear. Notably, our study only included prevalent cases linked to the Hospital Episodes Statistics database and, unlike Hwee et al,^5^ excluded diagnoses of other anti-neutrophil cytoplasmic antibody (ANCA)-associated vasculitis (eg, granulomatosis with polyangiitis, microscopic polyangiitis). Additionally, the 2014 British Society of Rheumatology guidelines^33^ in effect during the study (newer guidelines have since been published^22^) may have impacted how such patients were diagnosed/managed in practice and coded in the database in subsequent years. There were no relevant differences by sex in EGPA incidence and prevalence, in line with previous data.^3^

The median time from the first major EGPA symptom to diagnosis was almost 4 years (44 months), aligning with previous reports of 4 to 12 years.^17^^,^^30^^,^^32^^,^^34^ Collectively, these studies emphasize the need for greater awareness and earlier diagnosis of EGPA. A confirmed EGPA diagnosis can be challenging due to its rarity, its heterogeneous presentation, its overlap with other diseases, and the lack of universally accepted diagnostic criteria.^1^^,^^2^^,^^35^ Specialist consultations generally increased after diagnosis, with respiratory specialists frequently seen before and after EGPA diagnosis, reflecting the fact that most patients with EGPA have asthma or airway symptoms.^1^^,^^2^ After diagnosis, rheumatologists and respiratory and renal specialists were most frequently consulted, highlighting the need for multidisciplinary, coordinated clinical care.

To our knowledge, this is the first study to report EGPA-related mortality in clinical practice in England. The observed mortality rate was over 3 times higher than that reported in a global systematic literature review of 11 studies (10.63 [95% CI: 7.20-15.71] per 1000 PY).^9^ In our study, patients with EGPA had a death rate more than double (SMR of 2.3) that of the general population and comparable to an SMR of 1.6 reported in a Korean study based on data from a National Health Insurance database.^36^ This elevated SMR highlights the significant health risks associated with EGPA, emphasizing the need for greater awareness, earlier diagnosis, and targeted interventions to improve patient outcomes. Circulatory/cardiovascular diseases were the leading cause of death, aligning with the literature,^9^^,^^36^ and suggesting that early evaluation of cardiac involvement in EGPA and more effective treatments earlier in the patient pathway may be needed.

When reporting mortality data stratified by FFS, we used both versions of the FFS due to the inclusion of age >65 years as a criterion in the 2009 version. As a result, elderly patients, irrespective of organ involvement, automatically receive an FFS of 1, leading to potential bias in the analysis.

OGCs were the most frequently prescribed treatment in our study, followed by immunosuppressants, which is consistent with 2023 evidence-based guidelines.^21^ Updated 2025 guidelines recommend new treatments to reduce OGC reliance.^22^ The median daily OGC dose was relatively high (14.3 mg/day). Given the substantial toxicity associated with OGC use (most commonly skin atrophy, osteoporosis, and myopathy),^24^ treatment guidelines recommend tapering to the minimum effective dose.^22^ However, our study suggests that steroid tapering remains challenging; nearly one-half of patients experienced "other/unsuccessful" tapering outcomes, and only 27.0% achieved successful tapering. These findings underscore the need for improved OGC-sparing strategies to reduce toxicity risk.

The clinical outcomes in our study demonstrate a substantial disease burden. Nearly 10% of patients experienced major EGPA manifestations, and over 20% had nonmajor EGPA manifestations in the 6 months post-ID, reflecting challenges in achieving early disease control. Organ damage was present in 28.6% of patients during the 49-to-60–month period post-ID, predominantly ocular, ENT, pulmonary, and musculoskeletal damage. Remission rates remained low, with 60% of patients relapsing after achieving remission, consistent with EGPA’s relapsing nature.^1^^,^^2^ Vasculitic relapse was the most common type of relapse at any time post-ID. These findings emphasize the limitations of current EGPA management in England and the need for new and more effective treatments, especially for vasculitic/nonairway manifestations. Recently updated guidelines released by the British Society for Rheumatology now recommend anti-IL-5/receptor-α therapies for non–life- and non–organ-threatening disease to reduce OGC morbidity.^22^ There is increasing evidence that effective management of EGPA can be achieved through collaboration among multidisciplinary teams. Due to the wide range of symptoms associated with this condition and the potential for misdiagnosis due to similarities with other eosinophilic diseases,^37^ it is crucial for health care professionals from different specialties to work together to ensure optimal treatment.^21^

This study has several limitations. First, the incidence and prevalence of EGPA may be underestimated, potentially due to incorrect coding. Treatment data from specialist outpatient or hospital-administered settings (eg, biologics or treatments for severe EGPA, such as cyclophosphamide or rituximab) were unavailable in the dataset; therefore, care should be taken when interpreting the results on treatment patterns. Also, because no biologics were approved specifically for EGPA in the United Kingdom during the study period, biologic use could not be fully described and was substantially under-reported. The CPRD dataset records prescribed medications but lacks information on prescription reasons (eg, short-term vs maintenance therapy), the indication for prescribing (whether oral OGCs were given for EGPA or another concomitant condition), or whether medications were actually dispensed or taken by patients. We assumed full adherence to prescribed regimens, although this is unlikely in practice. Multiple clinical outcomes related to EGPA manifestations and organ damage were captured based on administrative health records using code lists that have not been previously validated in administrative databases. Assessments and laboratory values such as inflammatory biomarkers, allergic status, and ANCAs were unavailable, which is a key limitation because asthma management differs by inflammatory phenotype and EGPA manifestations may differ by ANCA status.^21^ Other clinical outcomes, including the FFS and health states, were defined using code proxies and algorithms developed for this study but lack prior validation. Therefore, the definitions of remission, stable disease, and relapse may not accurately reflect physician assessments. Additionally, because stable disease was defined as neither remission nor relapse, it likely captured a very heterogenous population of patients, including both refractory and nonrefractory patients. Any information prior to the start of the database is missing. As a result, there is no precise data regarding the age of patients diagnosed with asthma, or their history of respiratory diseases that may have caused the onset of wheezing.

### Conclusion

Our study underscores the significant health care and treatment burden of EGPA in England. EGPA management is complex and relies mainly on glucocorticoids and immunosuppressants, which may increase the risk of drug toxicity and associated complications. As such, there is an urgent and critical need for improved diagnostic pathways and more effective new treatments.

### Data sharing statement

This study is based in part on data from the Clinical Practice Research Datalink obtained under license from the UK Medicines and Healthcare products Regulatory Agency and so are not publicly available. The data are provided by patients and collected by the National Health Service as part of their care and support. The interpretation and conclusions contained in this study are those of the authors alone.

Data copyright ©2025, re-used with the permission of The Health and Social Care Information Centre. All rights reserved.

## Disclosure statement

This study was sponsored by AstraZeneca. S.S. is supported by the National Institute for Health and Care Research Imperial Biomedical Research Centre.

Disclosure of potential conflict of interest: S. Siddiqui has received speaker fees from GSK, AstraZeneca, Chiesi, Areteia Therapeutics, and Medscape; participates on advisory boards for AstraZeneca, Chiesi, GSK, Areteia Therapeutics, Sanofi, Lilly, and Kymera Therapeutics. B. Ding, P. Dolin, C. Edmonds, P. Jain, J. Rowell, L. Westerink, and S.Y. Chen are/were employees or contractors of AstraZeneca at the time of study conduct and may own stock/stock options. A. Lacetera, P. Suárez-Sánchez, C. Ariti, B. Podmore, and A. Kitchin Velarde are employees of OXON Epidemiology, which received funding from AstraZeneca to conduct the study.

The views expressed are those of the author(s) and not necessarily those of the National Institute for Health and Care Research or the Department of Health and Social Care.

## Supplementary data

Supplementary Tables E1-E11 and Figure E1 and E2

## References

[1] Fijolek J., Radzikowska E. (2023). Eosinophilic granulomatosis with polyangiitis—advances in pathogenesis, diagnosis, and treatment. Front Med (Lausanne).

[2] White J.P.E., Dubey S. (2023). Eosinophilic granulomatosis with polyangiitis: a review. Autoimmun Rev.

[3] Jakes R.W., Kwon N., Nordstrom B., Goulding R., Fahrbach K., Tarpey J. (2021). Burden of illness associated with eosinophilic granulomatosis with polyangiitis: a systematic literature review and meta-analysis. Clin Rheumatol.

[4] Bell C.F., Blauer-Peterson C., Mao J. (2021). Burden of illness and costs associated with eosinophilic granulomatosis with polyangiitis: evidence from a managed care database in the United States. J Manag Care Spec Pharm.

[5] Hwee J., Harper L., Fu Q., Nirantharakumar K., Mu G., Jakes R.W. (2024). Prevalence, incidence and healthcare burden of eosinophilic granulomatosis with polyangiitis in the UK. ERJ Open Res.

[6] Mahr A., Guillevin L., Poissonnet M., Aymé S. (2004). Prevalences of polyarteritis nodosa, microscopic polyangiitis, Wegener's granulomatosis, and Churg-Strauss syndrome in a French urban multiethnic population in 2000: a capture-recapture estimate. Arthritis Rheum.

[7] Mohammad A.J., Jacobsson L.T.H., Mahr A.D., Sturfelt G., Segelmark M. (2007). Prevalence of Wegener's granulomatosis, microscopic polyangiitis, polyarteritis nodosa and Churg–Strauss syndrome within a defined population in southern Sweden. Rheumatology (Oxford).

[8] Jakes R.W., Kwon N., Huynh L., Hwee J., Baylis L., Alfonso-Cristancho R. (2024). Burden of eosinophilic granulomatosis with polyangiitis in Europe. ERJ Open Res.

[9] Dolin P., Lucas S., Gamble A., Turner M., Rowell J. (2025). Systematic literature review and meta-analysis of the epidemiology and clinical burden of eosinophilic granulomatosis with polyangiitis. Mod Rheumatol.

[10] Sada K.E., Suzuki T., Joksaite S., Ju S., Logie J., Mu G. (2024). Trends in prevalence, treatment use, and disease burden in patients with eosinophilic granulomatosis with polyangiitis in Japan: real-world database analysis. Mod Rheumatol.

[11] Cottin V., Bel E., Bottero P., Dalhoff K., Humbert M., Lazor R. (2016). Respiratory manifestations of eosinophilic granulomatosis with polyangiitis (Churg-Strauss). Eur Respir J.

[12] Garcia-Vives E., Rodriguez-Palomares J.F., Harty L., Solans-Laque R., Jayne D. (2021). Heart disease in eosinophilic granulomatosis with polyangiitis (EGPA) patients: a screening approach proposal. Rheumatology (Oxford).

[13] Pagnoux C., Mahr A., Cohen P., Guillevin L. (2005). Presentation and outcome of gastrointestinal involvement in systemic necrotizing vasculitides: analysis of 62 patients with polyarteritis nodosa, microscopic polyangiitis, Wegener granulomatosis, Churg-Strauss syndrome, or rheumatoid arthritis-associated vasculitis. Medicine (Baltimore).

[14] Samson M., Puéchal X., Devilliers H., Ribi C., Cohen P., Bienvenu B. (2014). Mononeuritis multiplex predicts the need for immunosuppressive or immunomodulatory drugs for EGPA, PAN and MPA patients without poor-prognosis factors. Autoimmun Rev.

[15] Durel C.A., Sinico R.A., Teixeira V., Jayne D., Belenfant X., Marchand-Adam S. (2021). Renal involvement in eosinophilic granulomatosis with polyangiitis (EGPA): a multicentric retrospective study of 63 biopsy-proven cases. Rheumatology (Oxford).

[16] Comarmond C., Pagnoux C., Khellaf M., Cordier J.F., Hamidou M., Viallard J.F. (2013). Eosinophilic granulomatosis with polyangiitis (Churg-Strauss): clinical characteristics and long-term followup of the 383 patients enrolled in the French Vasculitis Study Group cohort. Arthritis Rheum.

[17] Doubelt I., Cuthbertson D., Carette S., Chung S.A., Forbess L.J., Khalidi N.A. (2021). Clinical manifestations and long-term outcomes of eosinophilic granulomatosis with polyangiitis in North America. ACR Open Rheumatol.

[18] Samson M., Puechal X., Devilliers H., Ribi C., Cohen P., Stern M. (2013). Long-term outcomes of 118 patients with eosinophilic granulomatosis with polyangiitis (Churg-Strauss syndrome) enrolled in two prospective trials. J Autoimmun.

[19] Chung S.A., Langford C.A., Maz M., Abril A., Gorelik M., Guyatt G. (2021). 2021 American College of Rheumatology/Vasculitis Foundation guideline for the management of antineutrophil cytoplasmic antibody-associated vasculitis. Arthritis Rheumatol.

[20] Hellmich B., Sanchez-Alamo B., Schirmer J.H., Berti A., Blockmans D., Cid M.C. (2024). EULAR recommendations for the management of ANCA-associated vasculitis: 2022 update. Ann Rheum Dis.

[21] Emmi G., Bettiol A., Gelain E., Bajema I.M., Berti A., Burns S. (2023). Evidence-based guideline for the diagnosis and management of eosinophilic granulomatosis with polyangiitis. Nat Rev Rheumatol.

[22] Biddle K., Jade J., Wilson-Morkeh H., Adikari M., Yaghchi C.A., Anastasa Z. (2025). The 2025 British Society for Rheumatology management recommendations for ANCA-associated vasculitis. Rheumatology (Oxford).

[23] Kamide Y., Taniguchi M. (2025). Eosinophilic granulomatosis with polyangiitis: current status and future perspectives. Respir Investig.

[24] Scherbacher P.J., Hellmich B., Feng Y.S., Löffler C. (2024). Prospective study of complications and sequelae of glucocorticoid therapy in ANCA-associated vasculitis. RMD Open.

[25] FVSG (2020). The French Vasculitis Study Group (FVSG) Registry. https://fairvasc.eu/the-fvsg-french-vasculitis-study-group-fvsgregistry/.

[26] Durel C.A., Berthiller J., Caboni S., Jayne D., Ninet J., Hot A. (2016). Long-term followup of a multicenter cohort of 101 patients with eosinophilic granulomatosis with polyangiitis (Churg-Strauss). Arthritis Care Res (Hoboken).

[27] Mukhtyar C., Lee R., Brown D., Carruthers D., Dasgupta B., Dubey S. (2009). Modification and validation of the Birmingham Vasculitis Activity Score (version 3). Ann Rheum Dis.

[28] Exley A.R., Bacon P.A., Luqmani R.A., Kitas G.D., Gordon C., Savage C.O. (1997). Development and initial validation of the Vasculitis Damage Index for the standardized clinical assessment of damage in the systemic vasculitides. Arthritis Rheum.

[29] Jardel S., Puéchal X., Le Quellec A., Pagnoux C., Hamidou M., Maurier F. (2018). Mortality in systemic necrotizing vasculitides: a retrospective analysis of the French Vasculitis Study Group registry. Autoimmun Rev.

[30] Moosig F., Bremer J.P., Hellmich B., Holle J.U., Holl-Ulrich K., Laudien M. (2013). A vasculitis centre based management strategy leads to improved outcome in eosinophilic granulomatosis and polyangiitis (Churg-Strauss, EGPA): monocentric experiences in 150 patients. Ann Rheum Dis.

[31] Berti A., Volcheck G.W., Cornec D., Smyth R.J., Specks U., Keogh K.A. (2018). Severe/uncontrolled asthma and overall survival in atopic patients with eosinophilic granulomatosis with polyangiitis. Respir Med.

[32] Puan Y., Ong K.Y., Tiew P.Y., Wen Chen G.X., Teo N.W.Y., Low A.H.L. (2025). Characteristics of severe asthma clinic patients with eosinophilic granulomatosis with polyangiitis. J Allergy Clin Immunol Pract.

[33] Ntatsaki E., Carruthers D., Chakravarty K., D'Cruz D., Harper L., Jayne D. (2014). BSR and BHPR guideline for the management of adults with ANCA-associated vasculitis. Rheumatology (Oxford).

[34] Puan Y., Tiew P.Y., Ong K.Y., Koh M.S. (2022). Diagnosis and management of eosinophilic granulomatosis with polyangiitis: perspective from the severe asthma clinic. Eur Respir J.

[35] Trivioli G., Terrier B., Vaglio A. (2020). Eosinophilic granulomatosis with polyangiitis: understanding the disease and its management. Rheumatology (Oxford).

[36] Lee J.H., Hong S.H., Yu I., Chang M.S., Park S., Lee S.J. (2024). Incidence, prevalence, and mortality of eosinophilic granulomatosis with polyangiitis in Korea: a nationwide population-based study. Allergy Asthma Immunol Res.

[37] Holle J.U., Vaglio A. (2025). Highlights from the plenary session: eosinophilic granulomatosis with polyangiitis and hypereosinophilic syndrome. Rheumatology (Oxford).

