# Supplementary material for: Epidemiology, clinical management, and outcomes in patients with eosinophilic granulomatosis with polyangiitis in England: A retrospective observational cohort study
Source: J Allergy Clin Immunol Glob. 2026 Jun 1;5(5):100740. doi: 10.1016/j.jacig.2026.100740 (PMC13332008; doi:10.1016/j.jacig.2026.100740)
Supplement: Supplementary Tables E1-E11 and Figure E1 and E2 [file mmc1.docx]

**Online Repository Material**

**Supplementary methods**

*Five factor score parameters (*1996 and 2009 versions*)*

The parameters assessed in the FFS 1996 version included proteinuria, creatininemia, severe gastrointestinal (GI) involvement (acute pancreatitis, chronic pancreatitis, GI bleed or perforation, GI ischemia or infarction), cardiomyopathy, and central nervous system (CNS) involvement (cerebrovascular accident, meningitis, organic confusion, seizure). In the FFS 2009 version, the following parameters were included: age >65 years, creatinine peak ≥150 µmoles/L (Stage IIIb–V chronic kidney disease [CKD], end-stage renal disease or dialysis), severe GI involvement (acute pancreatitis, chronic pancreatitis, GI bleed or perforation, GI ischemia or infarction), cardiac involvement (insufficiency, cardiomyopathy), and absence of ear, nose, and throat (ENT) involvement (allergic rhinitis, nasal polyps, or acute or chronic sinusitis).

*Mortality and survival*

The primary causes of death with >5 cases were assessed; the most common causes of death individually and aggregated by International Classification of Diseases (ICD) chapters were described. Cause-specific Standardized Mortality Ratios (SMRs) by most common ICD-10 chapters and a pooled "others" chapter were calculated for the overall incident cohorts and stratified by the FFS 1996 and 2009 versions and by health status. Mortality rates and 95% confidence intervals (CIs) were presented for each calendar year.

For SMR calculations, expected deaths were based on data from the Office for National Statistics (ONS) mortality statistics age- and sex-specific mortality rates 2010–2019. For the overall SMR, the 2011 ONS calculated age- and sex-specific mortality rates were used. The ICD-10 chapter-specific SMRs were calculated using the 2011 ICD-10 chapter-specific deaths and the 2011 mid-point ONS population estimates to calculate the ICD-10 chapter-specific mortality rates.^1^

*Prescribing rates*

Prescribing rates were defined as the total number of valid prescriptions within a period divided by the person time of follow-up within that period. Eosinophilic granulomatosis with polyangiitis (EGPA) medication included immunosuppressants (azathioprine, cyclophosphamide, mycophenolate mofetil, methotrexate), OGCs (prednisone, prednisolone, methylprednisolone), and non-prophylactic intravenous (IV)/intramuscular (IM) corticosteroids (methylprednisolone).

*Tapering*

To qualify for tapering during follow-up, a patient must have had a maximum OGC prescription of >10 mg/day between 30 days post-index date (ID) and 120 days before the end of follow-up. The date and dosage of this maximum prescription mark the start of the tapering episode and allow for the classification of the patient into one of four categories: successfully tapered (reduction to ≤4 mg/day or discontinued within 60 days after the start of tapering and remained on 0–4 mg/day for at least a further 60 days), partially tapered (≥50% reduction in OGC dose, but still above 4.0 mg/day within 60 days after the start of tapering and no subsequent increase in dose for at least a further 60 days), not tapered (no reduction within 60 days), and unsuccessful tapering (none of the previous categories).

*Type of relapse*

Among patients in relapse, the type(s) of relapse were classified as asthma relapse (new onset asthma in the time period prior to timepoint), nasal polyp relapse (new onset nasal polyposis in the time period prior to timepoint), vasculitic relapse (new EGPA event, except asthma or nasal polyp, in the time period prior to timepoint), or EGPA hospitalization relapse (hospitalized for EGPA or an EGPA event as the primary diagnosis in the time period prior to timepoint). A patient may have simultaneously had more than one type of relapse.

**Statistical analysis**

All statistical analyses were descriptive and performed using SAS statistical software, version 9.4 or higher. All variables were summarized descriptively through tabular and graphical displays. Continuous variables were summarized with the mean, standard deviation (SD), median, interquartile range, and minimum (min) and maximum (max) values. Categorical variables were summarized by categories with frequency counts and percentages. When applicable, 95% CIs were calculated.

*Prevalence*

Crude prevalence per 100,000 people and 95% CIs were provided for the total study period and for each study year (from 2006 to 2019). These results were presented aggregated and by age and sex strata.

The identification of prevalence started with identifying prevalent cases at the study initiation. Medical codes for diagnosis of EGPA were identified. Patients were considered prevalent cases when they had at least one EGPA diagnosis code identified through general practice (GP) visits or inpatient admissions before or during the study period. Prevalent cases were required to have Hospital Episodes Statistics linkage.

The prevalences of EGPA were calculated as: 100,000 times the number of individuals with EGPA during the period divided by the total number of individuals contributing time to the period. The denominator consisted of those patients who had an acceptable quality of data flag. 95% CIs were calculated using the Clopper-Pearson method.

*Incidence rates*

Crude incidence rates per 1,000,000 person-years and 95% CIs were provided for the total study period and for each study year. These results were presented aggregated and by age and sex strata.

Incidence cases were identified as patients with EGPA with their date of first diagnosis code within the study period to be considered as the ID for the incidence. To avoid overestimation of incident diagnoses, individuals were required to have at least 12 months of available data prior to the ID to be included in the calculation of incidence rates.

The incidence rates of EGPA were calculated as 1,000,000 times the number of incident cases identified in the period (date of first diagnosis record within the period) divided by the total follow-up time contributed by the reference population in that year.

The 95% CIs for the incidence rates were calculated from the Chi-square distribution values as follows:

$$LL=\frac{({\chi^{2}}_{\frac{\alpha}{2},2d})}{2}\mathrm{UL}=\frac{({\chi^{2}}_{1- \frac{\alpha}{2},2\left( d+1 \right)})}{2}$$

where LL and UL are lower and upper confidence limits respectively for the rate as d events per unit time exposed, $\chi_{a,v}^{2}$is the ${(100a)}^{\mathrm{th}}$ chi-square centile with ν degrees of freedom, where d is the number of observed incident cases.

**Reference**

**1**.https://www.ons.gov.uk/file?uri=/peoplepopulationandcommunity/birthsdeathsandmarriages/deaths/datasets/deathsregisteredinenglandandwalesseriesdrreferencetables/2019/finalreftables2019.xlsxTreatment patterns

**Supplementary tables**

**Table E1.** Codes to identify the patients with EGPA

| **Coding dictionary** | **Code** | **Term** |
| --- | --- | --- |
| ICD -10 (HES APC) | M30.1 | Polyarteritis with lung involvement [Churg-Strauss] |
| **Coding dictionary** | **SNOMED CT Concept ID** | **Term** |
| SNOMED (CPRD Aurum) | 82275008 | Allergic granulomatosis angiitis (All included terms below)  - Churg-Strauss syndrome  - Allergic granulomatous angiitis  - Eosinophilic granulomatosis with polyangiitis  - EGPA - eosinophilic granulomatosis with polyangiitis  - Allergic granulomatosis angiitis  - Churg Strauss syndrome  - Eosinophilic granulomatosis with polyangiitis  - CSS - Churg-Strauss syndrome |
|  | 317931000119101 | Pulmonary disease due to allergic granulomatosis angiitis (disorder) (All included terms below)  - Pulmonary disease due to allergic granulomatosis angiitis  - Churg Strauss polyarteritis with pulmonary involvement |

APC, Admitted Patient Care; CPRD, Clinical Practice Research Datalink; EGPA, eosinophilic granulomatosis with polyangiitis; HES, Hospital Episodes Statistics; ICD, International Classification of Diseases; SNOMED CT, Systematized Nomenclature of Medicine Clinical Terms.

**Table E2.** Five Factor Score characteristic codes

| **Characteristic** | **ICD-10-CM Code** |
| --- | --- |
| **Creatininemia^a^** |  |
| Stage IIIb–V chronic kidney disease, end-stage renal disease, or dialysis | I12.0, I13.11, I13.2, N18.32, N18.4, N18.5, N18.6, Z49, Z99.2 |
| **Severe GI involvement**:  Bowel perforation or bleeding (not due to appendicitis or cholecystitis) | K20.81, K20.91, K21.01, K22.11, K22.3, K25.0, K25.1, K25.2, K25.4, K25.5, K25.6, K26.0, K26.1, K26.2, K26.4, K26.5, K26.6, K27.0, K27.1, K27.2, K27.4, K27.5 K27.6, K28.0, K28.1, K28.2, K28.4, K28.5, K28.6, K29.01, K29.21, K29.31, K29.41, K29.51, K29.61, K29.71, K29.81, K29.91, K31.811, K35.2, K35.32, K35.33, K50.011, K50.111, K50.811, K50.911, K51.011, K51.211, K51.311, K51.411, K51.511, K51.811, K51.911, K55.21, K57.0, K57.11, K57.13, K57.2, K57.31, K57.33, K57.4, K57.51, K57.53, K57.8, K57.91, K57.93, K62.5, K63.1, K92.0, K92.1, K92.2 |
| GI infarction or ischemia | K55.0, K55.1, K55.8, K55.9 |
| Pancreatitis (acute or chronic) | K85.0, K85.1, K85.9, K86.1 |
| **Cardiac insufficiency**  Congestive Heart failure | I11.0, I13.0, I13.2, I26.0, I27.22, I50 |
| Cardiomyopathy | I25.5, I42.0, I42.1, I42.2, I42.3, I42.4, I42.5, I42.7, I42.8, I42.9, I43, O90.3 |
| **ENT involvement**  Allergic rhinitis | J30.1, J30.2, J30.5, J30.8, J30.9 |
| Nasal polyps | J33 |
| Acute or chronic sinusitis | J01, J32 |

^a^ For creatininemia other codes were included: HCPCS (G0049, G0052, G0257, G2170, G2171) and CPT (36818, 16819, 36820, 36825, 35830, 90935, 90937, 90945, 90947, 90960, 90961, 90962, 90963, 90966, 90970).

CPT, current procedural terminology; ENT, ear, nose, and throat; GI, gastrointestinal; HCPCS, healthcare common procedure coding system; ICD-10-CM, International Classification Of Diseases, Tenth Revision, Clinical Modification.

**Table E3.** Comorbidity diagnosis codes

| **Condition** | **ICD-10-CM Code** |
| --- | --- |
| Allergic rhinitis | J30.1, J30.2, J30.5, J30.8, J30.9 |
| Anxiety | F06.4, F40, F41, F43.1 |
| Arterial thrombosis | I63.0, I63.3, I74 |
| Arrhythmia | I47, I48, I49 |
| Asthma | J45 |
| Atopic dermatitis, eczema | L20, L30.9 |
| Back pain (dorsalgia) | M54.0, M54.1, M54.4, M54.5, M54.6, M54.8, M54.9 |
| Bronchiectasis | J47.9 |
| Chronic obstructive pulmonary disease | J41, J42, J43, J44, J98.2 |
| Cystic fibrosis with pulmonary manifestations | E84.0 |
| Deep vein thromboembolism | I80.1, I80.2, I80.3, I81, I82.2, I82.3, I82.4, I82.5, I82.62, I82.72, I82.9 |
| Depression | F06.31, F06.32, F25.1, F31.3, F31.4, F31.5, F31.6, F31.7, F31.8, F31.9, F32, F33, F34.1, F41.8, F43.21, F43.23 |
| Diffuse eosinophilic fasciitis | M35.4 |
| Eosinophilic cellulitis | L98.3 |
| Eosinophilic colitis | K52.82 |
| Dyslipidemia | E78, E88.81 |
| Eosinophilic endomyocardial disease | I42.3 |
| Eosinophilic esophagitis | K20.0 |
| Eosinophilic gastritis or gastroenteritis | K52.81 |
| Eosinophilic pneumonia | J82.81 |
| Gastroesophageal reflux disease | K21 |
| Hypereosinophilic syndrome | D72.11 |
| Hyperthyroidism | E05 |
| Hypothyroidism | E03.9 |
| Insomnia | G47.09, G47.00, G47.01, G47.0 |
| Interstitial pulmonary disease | J84, D86.0, D86.2 |
| Irritable bowel syndrome | K58 |
| Ischemic heart disease | I20, I21, I22, I23, I24, I25 |
| Nasal polyps | J33 |
| Obesity | E66.0, E66.1, E66.2, E66.8, E66.9, Z68.3, Z68.4 |
| Obstructive sleep apnea | G47.33 |
| Pulmonary eosinophilia | J82 |
| Throat or chest pain | R07 |
| Vitamin D deficiency | E55 |

ICD-10-CM, International Classification of Diseases, Tenth Revision, Clinical Modification.

**Table E4.** EGPA manifestations and related ICD-10-CM diagnosis codes

| **Organ System** | **Manifestation** | **ICD-10-CM Code** |
| --- | --- | --- |
| **Major EGPA manifestation** |  |  |
| **Abdominal** | GI ischemia or infarction | K55.0, K55.1, K55.8, K55.9 |
| **Ocular** | Retinal change (retinal vasculitis, thrombosis, exudate or hemorrhage) | H34, H35.0, H35.2, H35.6, H35.7 |
| **ENT** | Sensorineural hearing loss | H90.3, H90.4, H90.5, H90.6, H90.7, H90.8, H90.A2, H90.A3 |
| **Chest** | Hemoptysis or alveolar hemorrhage | R04.2, R04.89 |
|  | Respiratory failure | J96 |
| **Cardiovascular** | Ischemic cardiac pain | I20.9 |
|  | Cardiomyopathy | I25.5, I42.0, I42.1, I42.2, I42.3, I42.4, I42.5, I42.7, I42.8, I42.9, I43, O90.3 |
|  | Congestive heart failure | I11.0, I13.0, I13.2, I26.0, I27.22, I50 |
| **Renal** | Hematuria | N02, R31 |
|  | Creatinine proxy: Stage IV–V chronic kidney disease, end-stage renal disease | G0049, G0052, G0257, G2170, G2171 |
| **Nervous system** | Cerebrovascular accident | I60, I61, I62, I63, I67.6, I68, I69 |
|  | Spinal cord lesion | G95.1 |
|  | Cranial nerve palsy | G51.0, G51.2, H49.0, H49.1, H49.2, H49.3 |
|  | Mononeuritis multiplex | G58.7 |
| **Non-major EGPA manifestations** |  |  |
| **General** | Myalgia | M79.1 |
|  | Arthralgia | M25.5 |
|  | Fever | R50.81, R50.9 |
| **Skin** | Purpura | D69.0, D69.2 |
|  | Skin ulcer | L97, L98.4, E08.621, E08.622, E09.621, E09.622, E10.621, E10.622, E11.621, E11.622, E13.621, E13.622 |
|  | Urticaria | L50 |
|  | Nodules  Erythema nodosum | R22  L52 |
| **Mucous membrane** | Mouth ulcers | K12.3 |
|  | Genital ulcers | N48.5, N76.5, N76.6 |
| **Ocular** | Adnexal inflammation | H57.89 |
|  | Proptosis | H05.2 |
|  | Scleritis or episcleritis | H15.0, H15.1 |
|  | Conjunctivitis, blepharitis or keratitis | H01.0, H10, H16 |
|  | Moderate/severe visual impairment or blindness | H54.0, H54.1, H54.2, H54.3, H54.7, H54.8 |
|  | Uveitis | H20 |
| **ENT** | Bloody nasal discharge, crusts, nasal ulcers or nasal granulomata | J34.0, J34.8 |
|  | Nasal polyps | J33 |
|  | Subglottic stenosis | J38.6 |
|  | Conductive hearing loss | H90.0, H90.1, H90.2, H90.6, H90.7, H90.8, H90.A1, H90.A3 |
|  | Acute or chronic sinusitis | J01, J32 |
|  |  |  |
| **Chest** | Asthma | J45.909 |
|  | Wheeze | R06.2 |
|  | Lung nodules or cavities | R91 |
|  | Pleural effusion or pleurisy/pleuritis | J90, J91.8, R09.1 |
|  | Endobronchial involvement | J98.09 |
|  |  |  |
| **Cardiovascular** | Valvular heart disease | I34, I35, I36, I37 |
|  | Pericarditis or pericardiectomy | I30.0, I30.8, I30.9, I31.0, I31.1, I31.9 |
| **Abdominal** | Peritonitis | K65 |
|  | Bloody diarrhea | R19.7, k92.2 |
| **Renal** | Hypertension | I10, I11, I12, I13, I14, I16, I67.4 |
|  | Proteinuria | R80.0, R80.1, R80.9 |
|  | Creatinine proxy: Stage II-III chronic kidney disease | I12.9, I13.0, I13.10, N18.2, N18.3, N18.9 |
| **Nervous system** | Headache | G44.0, G44.1, G44.5, G44.8, R51 |
|  | Meningitis | G03 |
|  | Organic confusion | R41.0 |
|  | Seizure | G40, R56.9 |
|  | Sensory peripheral neuropathy | R20.2 |

EGPA, eosinophilic granulomatosis with polyangiitis; ENT, ear, nose, and throat; GI, gastrointestinal; ICD-10-CM, International Classification of Diseases, Tenth Revision, Clinical Modification.

**Table E5.** Organ damage manifestations and related ICD-10-CM diagnosis codes

| **Organ system** | **Persistent organ damage** | | **ICD-10-CM Code** |
| --- | --- | --- | --- |
| **Musculoskeletal damage** | Osteoporosis or vertebral collapse  Avascular necrosis  Osteomyelitis | M48.5, M80, M81  M87.0, M87.8, M87.9  M86 | |
| **Ocular damage** | Cataract | H25, H26.0, H26.2, H26.4, H26.8. H26.9, H28 | |
| **ENT damage** | Hearing loss  Nasal blockage  Chronic sinusitis | H90, H91.2, H91.8, H91.9  J34.89  J01, J32 | |
| **Pulmonary damage** | Pulmonary hypertension  Pulmonary fibrosis  Pulmonary infarction or embolism  Pleural fibrosis  Asthma, chronic breathlessness, or impaired lung function | I27.0, I27.2  J84.10, J84.112  I26  J94.1  J45, R06.02, R94.2 | |
| **Cardiovascular damage** | Angina / angioplasty  Myocardial infarction  Valvular heart disease  Pericarditis or pericardiectomy  Hypertension requiring antihypertensive therapy | I20, I25.11  I21, I22, I23, I24.1  I34, I35, I36, I37  I30.0, I30.8, I30.9, I31.0, I31.1, I31.9  I10, I11, I12, I13, I14, I16, I67.4 | |
| **Peripheral vascular damage** | Atherosclerosis of extremities  Other peripheral vascular disease  Other venous embolism or thrombosis | I70.2, I70.3, I70.4, I70.5, I70.6, I70.7, I70.8, I70.9  I73  I82 | |
| **GI damage** | GI infarction or ischemia  Acute pancreatitis | K55.0, K55.1, K55.8, K55.9  K85.0, K85.1, K85.9 | |
| **Renal damage** | Stage III–V chronic kidney disease  Proteinuria | I12.0, I13.11, I13.2, N18.32, N18.4, N18.5, N18.6, Z49, Z99.2  R80.0, R80.1, R80.9 | |
| **Neuropsychiatric damage** | Cognitive impairment  Major psychosis | F01, F03, G31.84, I69.01, I69.11, I69.21, I69.31, I69.81, I69.91, R41.0, R41.8, R41.9, R47.01  F06.0, F06.1, F06.2, F20, F21, F22, F23, F24, F25, F28, F29, F32.3 F33.3, R44 | |
| **Skin damage** | Skin ulcers | L97, L98.4, E08.621, E08.622, E09.621, E09.622, E10.621, E10.622, E11.621, E11.622, E13.621, E13.622 | |
| **Other** | Thrombocytopenia  Leukopenia, agranulocytosis  Pancytopenia  Diabetes (type 1 or type 2) | D69.3, D69.4, D69.6  D70, D72.81  D61.81 | |
|  |  | E08, E09, E10, E11, E13 | |

ENT, ear, nose, and throat; GI, gastrointestinal; ICD-10-CM, International Classification of Diseases, Tenth Revision, Clinical Modification.

**Table E6.** Baseline demographics and clinical characteristics of the incident EGPA cohort

| **Factor** | **EGPA incident cohort**  **(N=486)** |
| --- | --- |
| **Age at index date (years)** |  |
| n (miss) | 486 (0) |
| Mean (SD) | 57.9 (15.2) |
| Median (Min; Max) | 60.0 (12.0; 91.0) |
| *Categorical, N (%)* |  |
| <35 years^a^ | 36 (7.4) |
| 35-49 years | 101 (20.8) |
| 50-64 years | 173 (35.6) |
| 65-79 years | 144 (29.6) |
| 80+ years | 32 (6.6) |
| **Sex, N (%)** |  |
| Male | 242 (49.8) |
| Female | 244 (50.2) |
| **Region, N (%)** |  |
| Northeast | 13 (2.7) |
| Northwest | 101 (20.8) |
| Yorkshire & the Humber | 14 (2.9) |
| East Midlands | 7 (1.4) |
| West Midlands | 78 (16.0) |
| East of England | 34 (7.0) |
| London | 83 (17.1) |
| Southeast | 105 (21.6) |
| Southwest | 51 (10.5) |
| **Ethnicity, N (%)** |  |
| White | 417 (88.3) |
| Asian | 31 (6.6) |
| Black | 17 (3.6) |
| Mixed/Other^b^ | 7 (1.5) |
| *Missing* | 14 |
| **Index of Multiple Deprivation score, N (%)** |  |
| Quintile 1 (least deprived) | 125 (25.7) |
| Quintile 2 | 108 (22.2) |
| Quintile 3 | 91 (18.7) |
| Quintile 4 | 83 (17.1) |
| Quintile 5 (most deprived) | 79 (16.3) |

^a^The <18 and 18–34 age groups were combined due to small numbers. The <18 age group had less than 5 patients; ^b^The Mixed ethnicity group was combined with the 'Other' ethnicity group due to small numbers.

EGPA, eosinophilic granulomatosis with polyangiitis; SD, standard deviation.

**Table E7.** Deaths in the incident EGPA cohort stratified by index year and FFS 1996 and 2009

| **Deaths** | **N (%)** | **Death rate per 1000 person-years (95% CI)** |
| --- | --- | --- |
| **All causes^a^** | 98 (20.2) ^b^ | 37.1 (30.1–45.2) |
| **Stratified by index year** | | |
| **2006-2010** | 51 (52.0) | 39.0 (29.0–51.3) |
| **2011-2015** | 34 (34.7) | 32.0 (22.1–44.6) |
| **2016-2019** | 13 (13.3) | 48.5 (25.8–83.0) |
| **Stratified by FFS 1996** | | |
| **FFS 1996 = 0** | 61 (62.2) | 28.3 (21.6–36.3) |
| **FFS 1996 = 1** | 27 (27.6) | 61.2 (40.3–89.0) |
| **FFS 1996 = 2** | 10 (10.2) | 239.0 (114.6–439.5) |
| **Stratified by FFS 2009** | | |
| **FFS 2009 = 0** | 10 (10.2) | 11.9 (5.7–21.8) |
| **FFS 2009 = 1** | 24 (24.5) | 20.3 (13.0–30.2) |
| **FFS 2009 = 2** | 64 (65.3) | 104.4 (80.4–133.3) |

^a^Only causes with an N ≥5 have been listed. ^b^The percentage has as a denominator the incident cohort number. The percentages below use all causes N as the denominator.

CI, confidence interval; EGPA, eosinophilic granulomatosis with polyangiitis; FFS, Five Factor Score.

**Table E8.** Standardized mortality ratio in the incident EGPA cohort stratified by health state at ID

| **Health state at ID** | **Observed deaths** | **Expected deaths*** | **SMR (95% CI)** |
| --- | --- | --- | --- |
| **Remission** | 14 | 9.32 | 1.50 (0.82–2.52) |
| **Not in Remission** | 84 | 32.9 | 2.55 (2.04–3.16) |
| **Stable disease** | 55 | 24.2 | 2.27 (1.71–2.96) |
| **Relapse** | 29 | 8.7 | 3.33 (2.23–4.79) |

*Source: ONS Mortality Statistics: Age- and sex-specific mortality rates 2010–2019. For the overall SMR, the 2011 ONS calculated age- and sex-specific mortality rates were used.

CI, confidence interval; EGPA, eosinophilic granulomatosis with polyangiitis; ID, index date; ONS, Office for National Statistics; SMR, standardized mortality ratio.

**Table E9.** Duration of OGC and immunosuppressant treatment episodes

|  | **Episodes initiated** | | |
| --- | --- | --- | --- |
|  | **12 months  pre-ID** | **12 months  post-ID** | **All pre and/or  post-ID** |
| **Immunosuppressants** |  |  |  |
| **N of patients with at least one episode** | **45** | **202** | **264** |
| **Duration of treatment episodes (months)** | | | |
| n of episodes | 87 | 454 | 2847 |
| Mean (SD) | 5.9 (12.4) | 6.3 (13.9) | 3.4 (8.1) |
| Median (Q1; Q3) | 1.6 (1.0; 5.5) | 1.8 (1.0; 4.2) | 1.5 (1.0; 2.8) |
| Min; Max | <0.1; 94.1 | <0.1; 146.3 | <0.1; 146.3 |
| **OGCs** |  |  |  |
| **Duration of treatment episodes (months)** | | | |
| n of episodes | 581 | 670 | 3634 |
| Mean (SD) | 5.7 (12.3) | 5.7 (9.7) | 4.8 (9.3) |
| Median (Q1; Q3) | 1.9 (1.0; 5.2) | 2.7 (1.0; 5.6) | 2.0 (1.0; 4.6) |
| Min; Max | <0.1; 127.1 | <0.1; 96.5 | <0.1; 135.8 |
| **Categorization of treatment episodes, n (%)** | | | |
| 1–5 days | 6 (1.0) | <5 | 24 (0.7) |
| 6–14 days | 34 (5.9) | 7 (1.0) | 92 (2.5) |
| ≥15 days | 541 (93.1) | 660 (98.5) | 3,518 (96.8) |
| **Number of patients with at least one episode, n (%)^a^** | | | |
| Any duration | 331 | 348 | 447 |
| 1–5 days | <5 | <5 | 19 (4.3) |
| 6–14 days | 27 (8.2) | 7 (2.0) | 65 (14.5) |
| ≥15 days | 318 (96.1) | 346 (99.4) | 441 (98.7) |
| **Number of episodes per patient (ratio)** | | | |
| Any duration | 1.8 | 1.9 | 8.1 |
| 1–5 days | <0.1 | <0.1 | <0.1 |
| 6–14 days | 0.1 | <0.1 | 0.2 |
| ≥15 days | 1.6 | 1.9 | 7.9 |
| **Average annual number of episodes by patient** | | | |
| Any duration | 1.8 | 1.9 | 1.4 |
| 1–5 days | <0.1 | <0.1 | <0.1 |
| 6–14 days | 0.1 | <0.1 | <0.1 |
| ≥15 days | 1.6 | 1.9 | 1.4 |

^a^Patients may fall into more than one category.

ID, index date; OGC, oral glucocorticoid; Q1, first quartile; Q3, third quartile; SD, standard deviation.

**Table E10.** EGPA medications before and after ID, and overall

| **EGPA medication^a^, N (%)** | **Total post-ID** | **12 months pre-ID** | **12 months post-ID** |
| --- | --- | --- | --- |
|  |  |  |  |
| Prednisolone | 182 (37.4) | 290 (59.7) | 197 (40.5) |
| Azathioprine - Prednisolone | 104 (21.4) | 25 (5.1) | 104 (21.4) |
| Methotrexate - Prednisolone | 45 (9.3) | 13 (2.7) | 44 (9.1) |
| Mycophenolate mofetil - Prednisolone | 35 (7.2) | 6 (1.2) | 25 (5.1) |
| No drug | 34 (7.0) | 142 (29.2) | 75 (15.4) |
| Azathioprine - Methotrexate - Prednisolone | 20 (4.1) | <5 | 7 (1.4) |
| Azathioprine - Mycophenolate mofetil - Prednisolone | 18 (3.7) | 0 (0.0) | 6 (1.2) |
| Azathioprine - Cyclophosphamide monohydrate –  Prednisolone | 7 (1.4) | <5 | 10 (2.1) |
| Methotrexate - Mycophenolate mofetil - Prednisolone | 7 (1.4) | 0 (0.0) | 0 (0.0) |
| Azathioprine - Methotrexate - Mycophenolate mofetil - Prednisolone | 5 (1.0) | 0 (0.0) | 0 (0.0) |

^a^Regimen was defined as an alphabetical order of the drugs used in each time period. No combination or sequential regimens have been defined, so the drugs in each regimen can have been used at the same time or sequentially within the same time period.

EGPA, eosinophilic granulomatosis with polyangiitis; ID, index date.

**Table E11.** Persistent organ damage post-ID in the incident EGPA cohort

|  | | **Time since ID (months)** | | | | | | |
| --- | --- | --- | --- | --- | --- | --- | --- | --- |
|  | **0–6** | | **7–12** | **13–18** | **19–24** | **25–36** | **37–48** | **49–60** |
| **Organ damage** | **N (%)** | | **N (%)** | **N (%)** | **N (%)** | **N (%)** | **N (%)** | **N (%)** |
| **Number of patients** | **486 (100)** | | **451 (100)** | **436 (100)** | **412 (100)** | **392 (100)** | **327 (100)** | **283 (100)** |
| **Persistent damage in at least 1 organ** | **14 (2.9)** | | **30 (6.7)** | **47 (10.8)** | **60 (14.6)** | **86 (21.9)** | **91 (27.8)** | **81 (28.6)** |
| **Musculoskeletal damage** | **0 (0.0)** | | **<5** | **<5** | **<5** | **11 (2.8)** | **13 (4.0)** | **13 (4.6)** |
| Avascular necrosis | 0 (0.0) | | 0 (0.0) | 0 (0.0) | 0 (0.0) | 0 (0.0) | 0 (0.0) | 0 (0.0) |
| Osteomyelitis | 0 (0.0) | | 0 (0.0) | 0 (0.0) | <5 | <5 | <5 | <5 |
| Osteoporosis or vertebral collapse | 0 (0.0) | | <5 | <5 | <5 | 10 (2.6) | 12 (3.7) | 12 (4.2) |
| **Skin damage** | **<5** | | **<5** | **<5** | **<5** | **<5** | **<5** | **<5** |
| Skin ulcer | <5 | | <5 | <5 | <5 | <5 | <5 | <5 |
| **Ocular damage** | **<5** | | **<5** | **11 (2.5)** | **16 (3.9)** | **21 (5.4)** | **23 (7.0)** | **19 (6.7)** |
| Cataract | <5 | | <5 | 11 (2.5) | 16 (3.9) | 21 (5.4) | 23 (7.0) | 19 (6.7) |
| **ENT damage** | **<5** | | **5 (1.1)** | **7 (1.6)** | **9 (2.2)** | **13 (3.3)** | **18 (5.5)** | **18 (6.4)** |
| Chronic sinusitis | <5 | | <5 | 6 (1.4) | 7 (1.7) | 11 (2.8) | 11 (3.4) | 12 (4.2) |
| Hearing loss | 0 (0.0) | | 0 (0.0) | 0 (0.0) | 0 (0.0) | 0 (0.0) | 5 (1.5) | 5 (1.8) |
| Nasal blockage | 0 (0.0) | | <5 | <5 | <5 | <5 | <5 | <5 |
| **Pulmonary damage** | **<5** | | **6 (1.3)** | **8 (1.8)** | **10 (2.4)** | **15 (3.8)** | **14 (4.3)** | **13 (4.6)** |
| Asthma, chronic breathlessness or impaired lung function | <5 | | 5 (1.1) | 6 (1.4) | 8 (1.9) | 10 (2.6) | 8 (2.4) | 9 (3.2) |
| Pulmonary fibrosis | 0 (0.0) | | <5 | <5 | <5 | <5 | 5 (1.5) | <5 |
| Pulmonary hypertension | 0 (0.0) | | 0 (0.0) | 0 (0.0) | 0 (0.0) | <5 | 0 (0.0) | 0 (0.0) |
| Pulmonary infarction or embolism | <5 | | <5 | <5 | <5 | <5 | <5 | <5 |
| **CV damage** | **<5** | | **<5** | **7 (1.6)** | **8 (1.9)** | **11 (2.8)** | **14 (4.3)** | **16 (5.7)** |
| Angina | 0 (0.0) | | 0 (0.0) | 0 (0.0) | 0 (0.0) | 0 (0.0) | 0 (0.0) | <5 |
| Hypertension | 0 (0.0) | | <5 | <5 | <5 | 6 (1.5) | 7 (2.1) | 9 (3.2) |
| Myocardial infarction | 0 (0.0) | | <5 | <5 | <5 | <5 | <5 | <5 |
| Pericarditis | <5 | | <5 | <5 | <5 | <5 | <5 | <5 |
| Valvular heart disease | 0 (0.0) | | 0 (0.0) | <5 | <5 | <5 | <5 | <5 |
| **Peripheral vascular damage** | **<5** | | **<5** | **<5** | **<5** | **5 (1.3)** | **6 (1.8)** | **<5** |
| Atherosclerosis of extremities | 0 (0.0) | | 0 (0.0) | 0 (0.0) | 0 (0.0) | 0 (0.0) | 0 (0.0) | 0 (0.0) |
| Other peripheral vascular disease | <5 | | <5 | <5 | <5 | <5 | <5 | <5 |
| Other venous embolism or thrombosis | 0 (0.0) | | 0 (0.0) | <5 | <5 | <5 | <5 | <5 |
| **GI damage** | **0 (0.0)** | | **0 (0.0)** | **<5** | **<5** | **<5** | **0 (0.0)** | **0 (0.0)** |
| Acute pancreatitis | 0 (0.0) | | 0 (0.0) | 0 (0.0) | 0 (0.0) | 0 (0.0) | 0 (0.0) | 0 (0.0) |
| GI ischemia or infarction | 0 (0.0) | | 0 (0.0) | <5 | <5 | <5 | 0 (0.0) | 0 (0.0) |
| **Renal damage** | **<5** | | **<5** | **<5** | **6 (1.5)** | **10 (2.6)** | **6 (1.8)** | **8 (2.8)** |
| Stage IIIb-V chronic kidney disease, end stage renal disease or dialysis | <5 | | <5 | <5 | <5 | 7 (1.8) | <5 | 5 (1.8) |
| Proteinuria | 0 (0.0) | | 0 (0.0) | <5 | <5 | <5 | <5 | <5 |
| **Neuropsychiatric damage** | **0 (0.0)** | | **0 (0.0)** | **0 (0.0)** | **0 (0.0)** | **<5** | **<5** | **0 (0.0)** |
| Cognitive impairment | 0 (0.0) | | 0 (0.0) | 0 (0.0) | 0 (0.0) | <5 | <5 | 0 (0.0) |
| Major psychosis | 0 (0.0) | | 0 (0.0) | 0 (0.0) | 0 (0.0) | 0 (0.0) | 0 (0.0) | 0 (0.0) |
| **Other damage** | **<5** | | **9 (2.0)** | **11 (2.5)** | **12 (2.9)** | **14 (3.6)** | **14 (4.3)** | **14 (4.9)** |
| Leukopenia or agranulocytosis | 0 (0.0) | | 0 (0.0) | 0 (0.0) | 0 (0.0) | 0 (0.0) | 0 (0.0) | 0 (0.0) |
| Pancytopenia | 0 (0.0) | | 0 (0.0) | 0 (0.0) | 0 (0.0) | 0 (0.0) | 0 (0.0) | 0 (0.0) |
| Thrombocytopenia | 0 (0.0) | | 0 (0.0) | 0 (0.0) | 0 (0.0) | 0 (0.0) | 0 (0.0) | 0 (0.0) |
| Diabetes mellitus and complications | <5 | | 9 (2.0) | 11 (2.5) | 12 (2.9) | 14 (3.6) | 14 (4.3) | 14 (4.9) |
| **Number of organs with damage (max 11)** |  | |  |  |  |  |  |  |
| n (miss) | 14 (0) | | 30 (0) | 47 (0) | 60 (0) | 86 (0) | 91 (0) | 81 (0) |
| Mean (SD) | 1.1 (0.3) | | 1.1 (0.3) | 1.1 (0.3) | 1.2 (0.4) | 1.2 (0.4) | 1.2 (0.5) | 1.3 (0.7) |
| Median (Q1; Q3) | 1.0 (1.0; 1.0) | | 1.0 (1.0; 1.0) | 1.0 (1.0; 1.0) | 1.0 (1.0; 1.0) | 1.0 (1.0; 1.0) | 1.0 (1.0; 1.0) | 1.0 (1.0; 1.0) |
| Min; Max | 1.0; 2.0 | | 1.0; 2.0 | 1.0; 2.0 | 1.0; 2.0 | 1.0; 3.0 | 1.0; 4.0 | 1.0; 5.0 |

CV, cardiovascular; EGPA, eosinophilic granulomatosis with polyangiitis; ENT, ear, nose, and throat; GI, gastrointestinal; ID, index date; Q1, first quartile; Q3, third quartile.

**Supplementary figures**

**Figure E1.** Study design


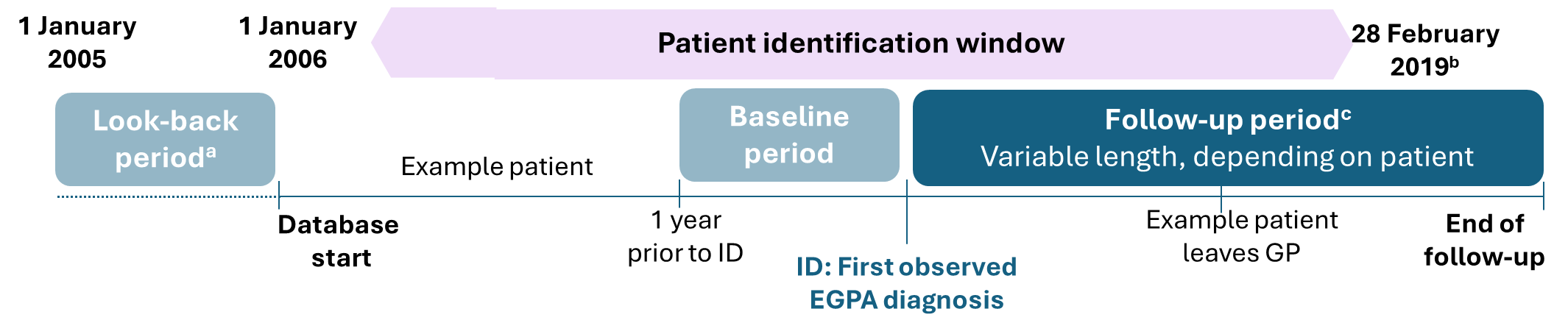


^a^The look-back period was up to 5 years before ID, at least 1 year prior to 2006, or at least 1 year prior to ID

^b^Patient inclusion ended on 28 February 2019; ^b^Follow-up was from ID to death, deregistration, last GP data collection, or study end on 28 February 2020.

EGPA, eosinophilic granulomatosis with polyangiitis; GP, general practice; ID, index date.

**Figure E2.** Patient flow diagram


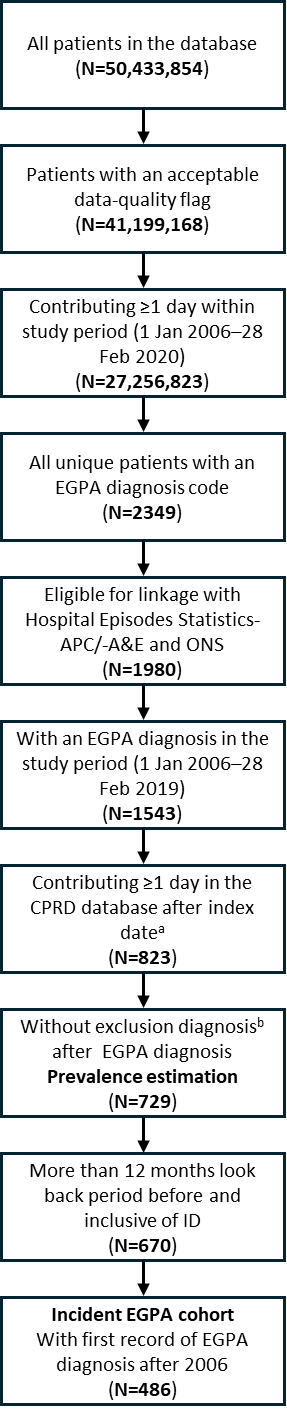


^a^Index date must be included in the CPRD validated period; ^b^Wegener's granulomatosis or GPA, MPA, polyarteritis nodosa, Takayasu arteritis, and giant cell arteritis.

A&E, accident & emergency; APC, admitted patient care; CPRD, Clinical Practice Research Datalink; EGPA, eosinophilic granulomatosis with polyangiitis; GPA, granulomatosis with polyangiitis; MPA, microscopic polyangiitis; ONS, Office for National Statistics.
